# Aqua­(di-2-pyridyl­amine-κ^2^ *N* ^2^,*N* ^2′^)(pyridine-2,6-dicarboxyl­ato-κ^3^ *O* ^2^,*N*,*O* ^6^)zinc monohydrate

**DOI:** 10.1107/S1600536811015571

**Published:** 2011-05-07

**Authors:** Figen Durkaya, Necmi Dege, Güneş Demirtaş, Ibrahim Uçar

**Affiliations:** a Department of Elementary Education, Elementary Science Education, Faculty of Education, Kırıkkale University, 71450 Yahşihan/Kırıkkale, Turkey; b Department of Physics, Faculty of Arts and Sciences, Ondokuz Mayıs University, 55139 Samsun, Turkey

## Abstract

In the title compound, [Zn(C_7_H_3_NO_4_)(C_10_H_9_N_3_)(H_2_O)]·H_2_O, the Zn^II^ atom has a distorted octa­hedral coordination geometry. One of the water mol­ecules is coordinated with the Zn^II^ ion and this mol­ecule forms an O—H⋯O inter­action with the lattice water mol­ecule. The pyridine-2,6-dicarboxyl­ate ligand is almost planar (r.m.s. deviation = 0.0242 Å). In the crystal, C—H⋯O, C—H⋯N, O—H⋯O and N—H⋯O hydrogen bonds are present.

## Related literature

For the biological activity of 2,6-pyridine­dicarb­oxy­lic acid, see: Chung *et al.* (1971 ▶); Tang *et al.* (1968 ▶). For the crystal structures of pyridine-2,6-dicarboxyl­ate derivatives, see: Uçar *et al.* (2007*a* ▶,*b* ▶); Uçar *et al.* (2009 ▶); Cui *et al.* (2011 ▶). For C—H⋯O inter­actions, see: Desiraju & Steiner (1999 ▶). 
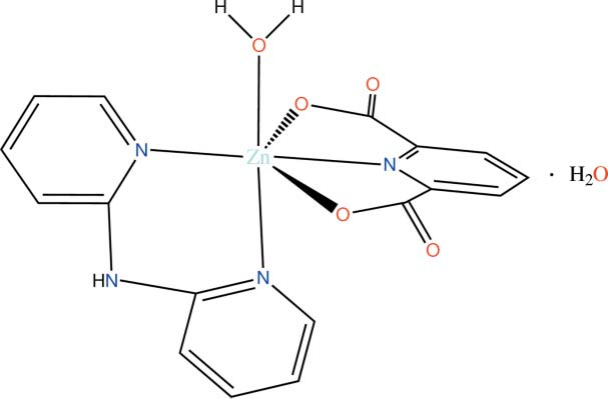


## Experimental

### 

#### Crystal data

[Zn(C_7_H_3_NO_4_)(C_10_H_9_N_3_)(H_2_O)]·H_2_O
*M* *_r_* = 437.71
Triclinic, 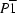

*a* = 6.8349 (5) Å
*b* = 11.1246 (8) Å
*c* = 12.1910 (9) Å
α = 96.109 (6)°
β = 96.404 (6)°
γ = 107.381 (6)°
*V* = 869.51 (11) Å^3^
*Z* = 2
Mo *K*α radiation
μ = 1.46 mm^−1^
*T* = 296 K
0.44 × 0.27 × 0.13 mm

#### Data collection

Stoe IPDS 2 diffractometer
Absorption correction: integration (*X-RED32*; Stoe & Cie, 2002 ▶) *T*_min_ = 0.598, *T*_max_ = 0.833
7786 measured reflections
3398 independent reflections
3093 reflections with *I* > 2σ(*I*)
*R*_int_ = 0.019

#### Refinement

*R*[*F* ^2^ > 2σ(*F* ^2^)] = 0.028
*wR*(*F* ^2^) = 0.071
*S* = 1.05
3398 reflections
315 parameters
3 restraints
H atoms treated by a mixture of independent and constrained refinement
Δρ_max_ = 0.36 e Å^−3^
Δρ_min_ = −0.51 e Å^−3^

### 

Data collection: *X-AREA* (Stoe & Cie, 2002 ▶); cell refinement: *X-AREA*; data reduction: *X-RED32* (Stoe & Cie, 2002 ▶); program(s) used to solve structure: *SHELXS97* (Sheldrick, 2008 ▶); program(s) used to refine structure: *SHELXL97* (Sheldrick, 2008 ▶); molecular graphics: *ORTEP-3 for Windows* (Farrugia, 1997 ▶); software used to prepare material for publication: *WinGX* (Farrugia, 1999 ▶) and *PLATON* (Spek, 2009 ▶).

## Supplementary Material

Crystal structure: contains datablocks I, global. DOI: 10.1107/S1600536811015571/zj2009sup1.cif

Structure factors: contains datablocks I. DOI: 10.1107/S1600536811015571/zj2009Isup2.hkl

Additional supplementary materials:  crystallographic information; 3D view; checkCIF report

## Figures and Tables

**Table 1:** Hydrogen-bond geometry (Å, °)

*D*—H⋯*A*	*D*—H	H⋯*A*	*D*⋯*A*	*D*—H⋯*A*
C10—H10⋯N4	0.93 (3)	2.59 (3)	3.189 (3)	123 (2)
O5—H5*B*⋯O6	0.82 (4)	1.95 (4)	2.750 (3)	166 (4)
N2—H5⋯O3^i^	0.75 (3)	2.08 (3)	2.823 (2)	172 (3)
O6—H6*B*⋯O1^ii^	0.86 (2)	2.05 (2)	2.832 (3)	151 (2)
O5—H5*A*⋯O2^iii^	0.83 (4)	1.97 (4)	2.804 (3)	176 (3)
C2—H2⋯O2^ii^	0.94 (3)	2.56 (3)	3.432 (3)	155 (2)
C3—H3⋯O4^iv^	0.86 (3)	2.41 (3)	3.117 (3)	140 (2)
C15—H15⋯O2^v^	0.88 (3)	2.57 (3)	3.309 (3)	142 (2)

Symmetry codes: (i) 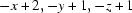; (ii) 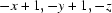; (iii) 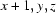; (iv) 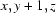; (v) 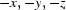.

**Fig. 1.**
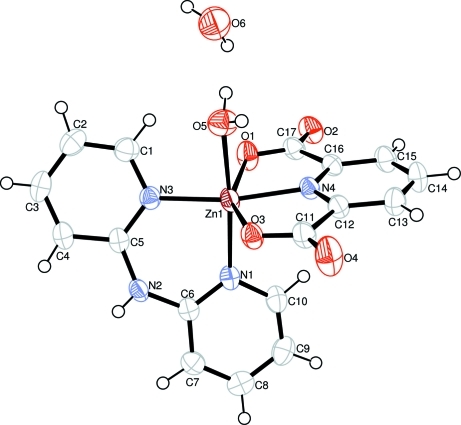
The asymmetric unit of the title compound, showing the atomic numbering scheme. Displacement ellipsoid are drawn at the 50% probability level.

**Fig. 2.**
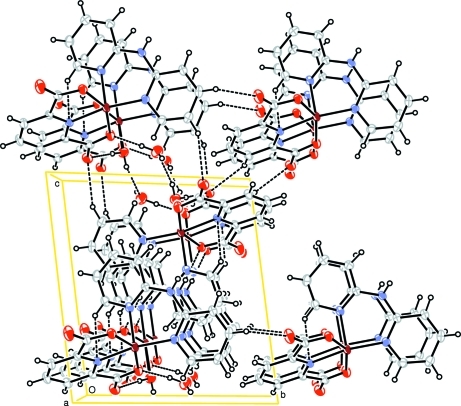
The crystal packing of the title compound in the unit cell. The hydrogen bonds were shown dashed lines.

## supplementary crystallographic information

### Comment

The large amount of the 2,6-pyridinedicarboxylic acid (dipicolinic acid, DPA) is contain by bacterial spores and may be related to heat resistance of bacterial spores (Chung *et al.*, 1971; Tang *et al.*, 1968).

In the Figure 1 is shown that the pyridine-2,6-dicarboxylato ligand has connected to Zn^II^ ion through the carboxyl group and ring nitrogen. The metal atom has also connected to 2,2-dipyridylamine ligand thought two ring nitrogens. Thus, the Zn(II) atom has a distorted octahedral coordination geometry by two N atoms from the dipyridylamine ligand, one N atom and two O atoms from the pyridine-2,6-dicarboxylato ligand and one O atom from aqua ligand.

The a lot of crystal structures with pyridine-2,6-dicarboxylato ligand were reported in literature ( Uçar *et al.*, 2007*a*; Uçar *et al.*, 2007*b*; Uçar *et al.*, 2009; Cui *et al.*, 2011) and our crystal structre is very similar with the crystal structure reported by Uçar *et al.*, (2007*a*). The Zn1—N1, Zn1—N3 and Zn1—N4 bond distances are 2.1318 (18) Å, 2.0327 (17) Å and 2.0269 (16) Å, respectively, while the Zn1—O1, Zn1—O3 and Zn1—O5 bond distances are 2.1947 (15) Å, 2.1755 (14) Å and 2.3148 (19) Å, respectively. In the crystal structure, the O3—C11—O4 and O1—C17—O2 bond angle for carboxylate groups are 126.5 (2)° and 126.4 (2)°, respectively. The some geometrical parameter were found as N1—Zn1—N3=89.01 (7)°, O3—Zn1—O1=152.19 (6)°, O1—Zn1—N4=75.75 (6)° and O3—Zn1—N4=76.80 (6)°.

The crystal structure contains intra and inter hydrogen bonds. The *d* parameter for C—H···O hydrogen bonds had separated a wide range and had different shapes for different donor atoms (Desiraju & Steiner, 1999). The *d* parameters of our crystal structure for H2···O2, H3···O4 and H15···O2 are 2.56 (3) Å, 2.41 (3) Å and 2.57 (3) Å, respectively. The *D* parameters for C—H···N interactions had given somewhat longer than C—H···O interactions (Desiraju & Steiner, 1999). The geometric parameters for C10—H10···N4 and C15—H15···O2 interactions are 0.93 (3) Å, 2.59 (3) Å, 3.189 (3) Å, 123 (2)° and 0.88 (3) Å, 2.57 (3) Å, 3.309 (3) Å, 142 (2)°, respectively. The *d* parameters for these interactions are 2.59 (3) Å and 2.57 (3) Å as well as *d* value for C10—H10···N4 is longer than *d* value for C15—H15···O2, but the *D* value is bigger for this interaction, due to the angle belong to C15—H15···O2 interaction is bigger than other interaction. The O5—H5B···O6 hydrogen bond is between the water molecules and the geometric parameters belong to this hydrogen bond are 0.82 (4) Å, 1.95 (4) Å, 2.750 (3) Å and 166 (4)°, respectively. The other hydrogen bonds have been given in Table 1.

### Experimental

To ethanol/water (30ml, ca. 1:1, v/v) containing ZnCl_2_.4H_2_O (1mmol) and disodium dipicolinate (1mmol), 2,2-dipyridylamine (1mmol) was added slowly with continuous stirring. The resulting solutions were refluxed for 1h and then filtered. The blue filtrates were allowed about 2 weeks at room temperature, and then the colorless crystals of title complex suitable for X-ray diffraction analyses were collected.

### Refinement

H6A and H6B atoms were located in a difference map and were refined with O–H and H···H distance restrains 0.80 (2) Å and 1.60 (2) Å, respectively. Other H atoms were located in a difference map and refined freely.

### Crystal data

**Table:**

[Zn(C_7_H_3_NO_4_)(C_10_H_9_N_3_)(H_2_O)]·H_2_O	*Z* = 2
*M**_r_* = 437.71	*F*(000) = 448
Triclinic, *P*1	*D*_x_ = 1.672 Mg m^−^^3^
Hall symbol: -P 1	Mo *K*α radiation, λ = 0.71073 Å
*a* = 6.8349 (5) Å	Cell parameters from 18443 reflections
*b* = 11.1246 (8) Å	θ = 2.4–27.4°
*c* = 12.1910 (9) Å	µ = 1.46 mm^−^^1^
α = 96.109 (6)°	*T* = 296 K
β = 96.404 (6)°	Prism, colorless
γ = 107.381 (6)°	0.44 × 0.27 × 0.13 mm
*V* = 869.51 (11) Å^3^	

### Data collection

**Table:**

Stoe IPDS 2 diffractometer	3398 independent reflections
Radiation source: fine-focus sealed tube	3093 reflections with *I* > 2σ(*I*)
graphite	*R*_int_ = 0.019
w–scan rotation	θ_max_ = 26.0°, θ_min_ = 2.4°
Absorption correction: integration (*X-RED32*; Stoe & Cie, 2002)	*h* = −8→8
*T*_min_ = 0.598, *T*_max_ = 0.833	*k* = −13→13
7786 measured reflections	*l* = −15→15

### Refinement

**Table:**

Refinement on *F*^2^	Primary atom site location: structure-invariant direct methods
Least-squares matrix: full	Secondary atom site location: difference Fourier map
*R*[*F*^2^ > 2σ(*F*^2^)] = 0.028	Hydrogen site location: inferred from neighbouring sites
*wR*(*F*^2^) = 0.071	H atoms treated by a mixture of independent and constrained refinement
*S* = 1.05	*w* = 1/[σ^2^(*F*_o_^2^) + (0.0354*P*)^2^ + 0.4854*P*] where *P* = (*F*_o_^2^ + 2*F*_c_^2^)/3
3398 reflections	(Δ/σ)_max_ = 0.001
315 parameters	Δρ_max_ = 0.36 e Å^−^^3^
3 restraints	Δρ_min_ = −0.51 e Å^−^^3^

### Special details

**Table:**

Geometry. All esds (except the esd in the dihedral angle between two l.s. planes) are estimated using the full covariance matrix. The cell esds are taken into account individually in the estimation of esds in distances, angles and torsion angles; correlations between esds in cell parameters are only used when they are defined by crystal symmetry. An approximate (isotropic) treatment of cell esds is used for estimating esds involving l.s. planes.
Refinement. Refinement of F^2^ against ALL reflections. The weighted R-factor wR and goodness of fit S are based on F^2^, conventional R-factors R are based on F, with F set to zero for negative F^2^. The threshold expression of F^2^ > 2sigma(F^2^) is used only for calculating R-factors(gt) etc. and is not relevant to the choice of reflections for refinement. R-factors based on F^2^ are statistically about twice as large as those based on F, and R- factors based on ALL data will be even larger.

### Fractional atomic coordinates and isotropic or equivalent isotropic displacement parameters (Å^2^)

**Table:**

	*x*	*y*	*z*	*U*_iso_*/*U*_eq_	
C1	0.8321 (4)	0.6257 (2)	0.2034 (2)	0.0405 (5)	
C2	0.9281 (4)	0.7536 (2)	0.2147 (2)	0.0457 (5)	
C3	1.0007 (4)	0.8203 (2)	0.3211 (2)	0.0448 (5)	
C4	0.9665 (3)	0.7576 (2)	0.4108 (2)	0.0384 (5)	
C5	0.8625 (3)	0.62585 (18)	0.39379 (17)	0.0305 (4)	
C6	0.7053 (3)	0.45086 (19)	0.50443 (17)	0.0301 (4)	
C7	0.6890 (3)	0.4313 (2)	0.61532 (19)	0.0371 (5)	
C8	0.5781 (4)	0.3143 (2)	0.6365 (2)	0.0428 (5)	
C9	0.4779 (4)	0.2187 (2)	0.5475 (2)	0.0457 (5)	
C10	0.4947 (4)	0.2455 (2)	0.4423 (2)	0.0421 (5)	
C11	0.8865 (3)	0.1646 (2)	0.28574 (18)	0.0373 (5)	
C12	0.6742 (3)	0.10009 (19)	0.21539 (17)	0.0318 (4)	
C13	0.5899 (4)	−0.0290 (2)	0.1784 (2)	0.0404 (5)	
C14	0.3928 (4)	−0.0736 (2)	0.1172 (2)	0.0451 (6)	
C15	0.2853 (3)	0.0102 (2)	0.0934 (2)	0.0400 (5)	
C16	0.3784 (3)	0.13814 (19)	0.13253 (16)	0.0315 (4)	
C17	0.2853 (3)	0.2443 (2)	0.11450 (17)	0.0353 (4)	
N1	0.6108 (3)	0.35941 (16)	0.41824 (15)	0.0340 (4)	
N2	0.8254 (3)	0.56958 (17)	0.48748 (16)	0.0328 (4)	
N3	0.8026 (3)	0.55946 (16)	0.29092 (14)	0.0319 (4)	
N4	0.5679 (3)	0.17928 (15)	0.19219 (14)	0.0301 (3)	
O1	0.4027 (2)	0.35555 (15)	0.15426 (14)	0.0452 (4)	
O2	0.1071 (2)	0.21538 (16)	0.06327 (14)	0.0456 (4)	
O3	0.9333 (2)	0.28411 (14)	0.31253 (12)	0.0360 (3)	
O4	0.9925 (3)	0.09778 (17)	0.31240 (19)	0.0629 (6)	
O5	0.8755 (3)	0.38070 (19)	0.10477 (16)	0.0480 (4)	
O6	0.7196 (4)	0.4353 (2)	−0.0951 (2)	0.0736 (6)	
Zn1	0.69556 (4)	0.36631 (2)	0.25541 (2)	0.03462 (9)	
H6A	0.598 (3)	0.411 (2)	−0.062 (2)	0.052*	
H6B	0.727 (4)	0.5068 (19)	−0.118 (2)	0.052*	
H1	0.788 (4)	0.577 (3)	0.131 (2)	0.046 (7)*	
H2	0.937 (4)	0.790 (3)	0.149 (2)	0.054 (8)*	
H3	1.064 (4)	0.901 (3)	0.331 (2)	0.051 (8)*	
H4	1.005 (4)	0.797 (2)	0.483 (2)	0.039 (6)*	
H5	0.881 (4)	0.612 (2)	0.540 (2)	0.032 (6)*	
H5A	0.941 (5)	0.329 (3)	0.094 (3)	0.060 (9)*	
H5B	0.818 (6)	0.384 (3)	0.043 (3)	0.077 (11)*	
H7	0.754 (4)	0.499 (3)	0.672 (2)	0.045 (7)*	
H8	0.567 (4)	0.299 (3)	0.709 (2)	0.046 (7)*	
H9	0.391 (5)	0.139 (3)	0.558 (2)	0.059 (8)*	
H10	0.427 (4)	0.185 (3)	0.380 (2)	0.047 (7)*	
H13	0.661 (4)	−0.083 (3)	0.195 (2)	0.052 (8)*	
H14	0.336 (4)	−0.159 (3)	0.092 (2)	0.053 (8)*	
H15	0.160 (5)	−0.016 (3)	0.055 (2)	0.058 (8)*	

### Atomic displacement parameters (Å^2^)

**Table:**

	*U*^11^	*U*^22^	*U*^33^	*U*^12^	*U*^13^	*U*^23^
C1	0.0449 (12)	0.0410 (12)	0.0389 (12)	0.0179 (10)	0.0067 (9)	0.0071 (10)
C2	0.0487 (13)	0.0418 (13)	0.0538 (14)	0.0191 (11)	0.0142 (11)	0.0175 (11)
C3	0.0397 (12)	0.0277 (11)	0.0670 (16)	0.0088 (9)	0.0094 (11)	0.0105 (11)
C4	0.0347 (11)	0.0291 (10)	0.0480 (13)	0.0097 (8)	−0.0003 (9)	−0.0016 (9)
C5	0.0232 (9)	0.0277 (10)	0.0405 (11)	0.0108 (7)	−0.0005 (8)	0.0015 (8)
C6	0.0235 (9)	0.0304 (10)	0.0367 (10)	0.0116 (8)	−0.0002 (8)	0.0020 (8)
C7	0.0329 (10)	0.0413 (12)	0.0373 (11)	0.0142 (9)	0.0016 (9)	0.0032 (9)
C8	0.0398 (12)	0.0497 (14)	0.0442 (13)	0.0184 (10)	0.0102 (10)	0.0136 (11)
C9	0.0403 (12)	0.0372 (12)	0.0614 (15)	0.0101 (10)	0.0162 (11)	0.0119 (11)
C10	0.0381 (12)	0.0329 (11)	0.0493 (13)	0.0046 (9)	0.0074 (10)	−0.0011 (10)
C11	0.0351 (11)	0.0342 (11)	0.0404 (11)	0.0128 (9)	−0.0054 (9)	0.0014 (9)
C12	0.0320 (10)	0.0295 (10)	0.0327 (10)	0.0106 (8)	0.0003 (8)	0.0011 (8)
C13	0.0405 (12)	0.0298 (11)	0.0490 (13)	0.0121 (9)	0.0000 (10)	0.0024 (9)
C14	0.0428 (12)	0.0269 (11)	0.0560 (14)	0.0028 (9)	0.0006 (11)	−0.0049 (10)
C15	0.0304 (11)	0.0361 (11)	0.0440 (12)	0.0029 (9)	−0.0040 (9)	−0.0027 (9)
C16	0.0277 (9)	0.0326 (10)	0.0299 (10)	0.0064 (8)	−0.0008 (8)	0.0002 (8)
C17	0.0310 (10)	0.0376 (11)	0.0344 (10)	0.0106 (9)	−0.0033 (8)	0.0009 (9)
N1	0.0306 (8)	0.0285 (8)	0.0397 (9)	0.0070 (7)	0.0025 (7)	0.0006 (7)
N2	0.0327 (9)	0.0276 (9)	0.0322 (9)	0.0067 (7)	−0.0051 (7)	−0.0022 (7)
N3	0.0318 (8)	0.0286 (8)	0.0350 (9)	0.0106 (7)	0.0016 (7)	0.0031 (7)
N4	0.0294 (8)	0.0277 (8)	0.0300 (8)	0.0078 (7)	−0.0021 (6)	−0.0001 (7)
O1	0.0388 (8)	0.0328 (8)	0.0579 (10)	0.0125 (7)	−0.0144 (7)	−0.0006 (7)
O2	0.0343 (8)	0.0477 (9)	0.0491 (9)	0.0145 (7)	−0.0125 (7)	−0.0026 (7)
O3	0.0324 (7)	0.0308 (7)	0.0396 (8)	0.0092 (6)	−0.0078 (6)	−0.0010 (6)
O4	0.0539 (11)	0.0424 (10)	0.0875 (14)	0.0246 (8)	−0.0250 (10)	−0.0019 (9)
O5	0.0541 (10)	0.0532 (11)	0.0450 (10)	0.0274 (9)	0.0081 (8)	0.0114 (8)
O6	0.0919 (17)	0.0610 (13)	0.0670 (14)	0.0290 (12)	−0.0116 (12)	0.0154 (11)
Zn1	0.03612 (14)	0.02497 (13)	0.03752 (15)	0.00839 (9)	−0.00719 (10)	−0.00130 (9)

### Geometric parameters (Å, °)

**Table:**

C1—N3	1.360 (3)	C11—C12	1.525 (3)
C1—C2	1.362 (3)	C12—N4	1.332 (3)
C1—H1	0.95 (3)	C12—C13	1.381 (3)
C2—C3	1.382 (4)	C13—C14	1.384 (3)
C2—H2	0.94 (3)	C13—H13	0.90 (3)
C3—C4	1.363 (4)	C14—C15	1.383 (3)
C3—H3	0.86 (3)	C14—H14	0.91 (3)
C4—C5	1.406 (3)	C15—C16	1.380 (3)
C4—H4	0.92 (3)	C15—H15	0.88 (3)
C5—N3	1.339 (3)	C16—N4	1.334 (2)
C5—N2	1.372 (3)	C16—C17	1.523 (3)
C6—N1	1.336 (3)	C17—O2	1.237 (2)
C6—N2	1.382 (3)	C17—O1	1.266 (3)
C6—C7	1.403 (3)	N1—Zn1	2.1318 (18)
C7—C8	1.364 (3)	N2—H5	0.75 (3)
C7—H7	0.93 (3)	N3—Zn1	2.0327 (17)
C8—C9	1.389 (4)	N4—Zn1	2.0269 (16)
C8—H8	0.93 (3)	O1—Zn1	2.1947 (15)
C9—C10	1.357 (4)	O3—Zn1	2.1755 (14)
C9—H9	0.94 (3)	O5—Zn1	2.3148 (19)
C10—N1	1.361 (3)	O5—H5A	0.83 (4)
C10—H10	0.93 (3)	O5—H5B	0.82 (4)
C11—O4	1.226 (3)	O6—H6A	0.944 (16)
C11—O3	1.268 (3)	O6—H6B	0.860 (17)
			
N3—C1—C2	123.8 (2)	C16—C15—C14	118.5 (2)
N3—C1—H1	116.2 (16)	C16—C15—H15	120 (2)
C2—C1—H1	119.9 (16)	C14—C15—H15	121.7 (19)
C1—C2—C3	118.3 (2)	N4—C16—C15	120.37 (19)
C1—C2—H2	116.5 (18)	N4—C16—C17	113.52 (17)
C3—C2—H2	125.2 (18)	C15—C16—C17	126.11 (18)
C4—C3—C2	119.4 (2)	O2—C17—O1	126.4 (2)
C4—C3—H3	120.3 (19)	O2—C17—C16	118.48 (19)
C2—C3—H3	120.4 (19)	O1—C17—C16	115.11 (17)
C3—C4—C5	119.7 (2)	C6—N1—C10	117.04 (19)
C3—C4—H4	123.9 (16)	C6—N1—Zn1	122.80 (14)
C5—C4—H4	116.5 (16)	C10—N1—Zn1	118.08 (15)
N3—C5—N2	122.07 (18)	C5—N2—C6	133.38 (18)
N3—C5—C4	121.2 (2)	C5—N2—H5	113.4 (19)
N2—C5—C4	116.70 (19)	C6—N2—H5	113.2 (19)
N1—C6—N2	120.82 (19)	C5—N3—C1	117.46 (18)
N1—C6—C7	122.11 (19)	C5—N3—Zn1	125.03 (14)
N2—C6—C7	117.06 (18)	C1—N3—Zn1	117.26 (15)
C8—C7—C6	119.2 (2)	C12—N4—C16	121.92 (17)
C8—C7—H7	122.3 (16)	C12—N4—Zn1	118.39 (13)
C6—C7—H7	118.4 (16)	C16—N4—Zn1	119.64 (14)
C7—C8—C9	119.1 (2)	C17—O1—Zn1	115.49 (13)
C7—C8—H8	120.6 (17)	C11—O3—Zn1	115.28 (12)
C9—C8—H8	120.2 (17)	Zn1—O5—H5A	117 (2)
C10—C9—C8	118.5 (2)	Zn1—O5—H5B	120 (3)
C10—C9—H9	119.6 (18)	H5A—O5—H5B	106 (3)
C8—C9—H9	121.8 (18)	H6A—O6—H6B	107 (2)
C9—C10—N1	123.9 (2)	N4—Zn1—N3	169.48 (7)
C9—C10—H10	121.3 (17)	N4—Zn1—N1	98.90 (7)
N1—C10—H10	114.8 (17)	N3—Zn1—N1	89.01 (7)
O4—C11—O3	126.5 (2)	N4—Zn1—O3	76.80 (6)
O4—C11—C12	118.25 (19)	N3—Zn1—O3	110.79 (6)
O3—C11—C12	115.21 (18)	N1—Zn1—O3	86.62 (6)
N4—C12—C13	120.53 (18)	N4—Zn1—O1	75.75 (6)
N4—C12—C11	114.27 (17)	N3—Zn1—O1	95.92 (6)
C13—C12—C11	125.19 (19)	N1—Zn1—O1	101.92 (7)
C12—C13—C14	118.4 (2)	O3—Zn1—O1	152.19 (6)
C12—C13—H13	120.5 (18)	N4—Zn1—O5	85.73 (7)
C14—C13—H13	121.1 (18)	N3—Zn1—O5	88.44 (7)
C15—C14—C13	120.2 (2)	N1—Zn1—O5	164.45 (7)
C15—C14—H14	120.7 (18)	O3—Zn1—O5	79.96 (7)
C13—C14—H14	119.1 (18)	O1—Zn1—O5	93.61 (7)
			
N3—C1—C2—C3	0.3 (4)	C15—C16—N4—Zn1	−176.87 (16)
C1—C2—C3—C4	−2.7 (4)	C17—C16—N4—Zn1	3.2 (2)
C2—C3—C4—C5	1.4 (3)	O2—C17—O1—Zn1	173.65 (19)
C3—C4—C5—N3	2.5 (3)	C16—C17—O1—Zn1	−6.8 (2)
C3—C4—C5—N2	−177.3 (2)	O4—C11—O3—Zn1	−176.9 (2)
N1—C6—C7—C8	1.9 (3)	C12—C11—O3—Zn1	1.7 (2)
N2—C6—C7—C8	−177.71 (19)	C12—N4—Zn1—N3	139.2 (3)
C6—C7—C8—C9	−2.3 (3)	C16—N4—Zn1—N3	−43.2 (4)
C7—C8—C9—C10	0.2 (4)	C12—N4—Zn1—N1	−82.39 (16)
C8—C9—C10—N1	2.5 (4)	C16—N4—Zn1—N1	95.14 (16)
O4—C11—C12—N4	178.7 (2)	C12—N4—Zn1—O3	1.98 (15)
O3—C11—C12—N4	0.0 (3)	C16—N4—Zn1—O3	179.51 (16)
O4—C11—C12—C13	−0.1 (4)	C12—N4—Zn1—O1	177.47 (17)
O3—C11—C12—C13	−178.9 (2)	C16—N4—Zn1—O1	−4.99 (15)
N4—C12—C13—C14	−0.2 (3)	C12—N4—Zn1—O5	82.65 (16)
C11—C12—C13—C14	178.5 (2)	C16—N4—Zn1—O5	−99.81 (16)
C12—C13—C14—C15	0.5 (4)	C5—N3—Zn1—N4	162.2 (3)
C13—C14—C15—C16	−0.3 (4)	C1—N3—Zn1—N4	−23.7 (5)
C14—C15—C16—N4	−0.2 (3)	C5—N3—Zn1—N1	23.17 (16)
C14—C15—C16—C17	179.7 (2)	C1—N3—Zn1—N1	−162.73 (16)
N4—C16—C17—O2	−177.65 (19)	C5—N3—Zn1—O3	−62.84 (17)
C15—C16—C17—O2	2.4 (3)	C1—N3—Zn1—O3	111.27 (16)
N4—C16—C17—O1	2.7 (3)	C5—N3—Zn1—O1	125.05 (16)
C15—C16—C17—O1	−177.2 (2)	C1—N3—Zn1—O1	−60.85 (16)
N2—C6—N1—C10	−179.72 (19)	C5—N3—Zn1—O5	−141.49 (16)
C7—C6—N1—C10	0.7 (3)	C1—N3—Zn1—O5	32.62 (16)
N2—C6—N1—Zn1	17.0 (3)	C6—N1—Zn1—N4	160.71 (15)
C7—C6—N1—Zn1	−162.58 (15)	C10—N1—Zn1—N4	−2.40 (17)
C9—C10—N1—C6	−3.0 (3)	C6—N1—Zn1—N3	−26.25 (16)
C9—C10—N1—Zn1	161.11 (19)	C10—N1—Zn1—N3	170.64 (17)
N3—C5—N2—C6	−10.9 (3)	C6—N1—Zn1—O3	84.64 (16)
C4—C5—N2—C6	168.9 (2)	C10—N1—Zn1—O3	−78.47 (16)
N1—C6—N2—C5	6.7 (3)	C6—N1—Zn1—O1	−122.10 (16)
C7—C6—N2—C5	−173.7 (2)	C10—N1—Zn1—O1	74.79 (17)
N2—C5—N3—C1	174.93 (19)	C6—N1—Zn1—O5	54.4 (3)
C4—C5—N3—C1	−4.8 (3)	C10—N1—Zn1—O5	−108.8 (3)
N2—C5—N3—Zn1	−11.0 (3)	C11—O3—Zn1—N4	−1.98 (15)
C4—C5—N3—Zn1	169.25 (15)	C11—O3—Zn1—N3	−174.37 (15)
C2—C1—N3—C5	3.6 (3)	C11—O3—Zn1—N1	97.98 (16)
C2—C1—N3—Zn1	−171.01 (19)	C11—O3—Zn1—O1	−11.4 (2)
C13—C12—N4—C16	−0.4 (3)	C11—O3—Zn1—O5	−89.92 (16)
C11—C12—N4—C16	−179.23 (18)	C17—O1—Zn1—N4	6.50 (16)
C13—C12—N4—Zn1	177.11 (17)	C17—O1—Zn1—N3	179.98 (17)
C11—C12—N4—Zn1	−1.8 (2)	C17—O1—Zn1—N1	−89.78 (17)
C15—C16—N4—C12	0.6 (3)	C17—O1—Zn1—O3	15.9 (3)
C17—C16—N4—C12	−179.36 (18)	C17—O1—Zn1—O5	91.17 (17)

### Hydrogen-bond geometry (Å, °)

**Table:**

*D*—H···*A*	*D*—H	H···*A*	*D*···*A*	*D*—H···*A*
C10—H10···N4	0.93 (3)	2.59 (3)	3.189 (3)	123 (2)
O5—H5B···O6	0.82 (4)	1.95 (4)	2.750 (3)	166 (4)
N2—H5···O3^i^	0.75 (3)	2.08 (3)	2.823 (2)	172 (3)
O6—H6B···O1^ii^	0.86 (2)	2.05 (2)	2.832 (3)	151 (2)
O5—H5A···O2^iii^	0.83 (4)	1.97 (4)	2.804 (3)	176 (3)
C2—H2···O2^ii^	0.94 (3)	2.56 (3)	3.432 (3)	155 (2)
C3—H3···O4^iv^	0.86 (3)	2.41 (3)	3.117 (3)	140 (2)
C15—H15···O2^v^	0.88 (3)	2.57 (3)	3.309 (3)	142 (2)

Symmetry codes: (i) −*x*+2, −*y*+1, −*z*+1; (ii) −*x*+1, −*y*+1, −*z*; (iii) *x*+1, *y*, *z*; (iv) *x*, *y*+1, *z*; (v) −*x*, −*y*, −*z*.
